# Accelerometer‐derived sleep measures in idiopathic dystonia: A UK Biobank cohort study

**DOI:** 10.1002/brb3.2933

**Published:** 2023-08-07

**Authors:** Grace A Bailey, Megan E. Wadon, Sandra Komarzynski, Clare Matthews, Elin Haf Davies, Kathryn J. Peall

**Affiliations:** ^1^ Neuroscience and Mental Health Research Institute Division of Psychological Medicine and Clinical Neurosciences Cardiff University School of Medicine Cardiff UK; ^2^ Aparito Limited Wrexham UK

**Keywords:** accelerometer, dystonia, sleep

## Abstract

**Background:**

Sleep disturbance is an increasingly recognized non‐motor trait in dystonia, with varying findings reported to date. Here, we examine sleep in a UK Biobank derived dystonia cohort using subjective self‐reported sleep symptoms and objective accelerometer‐derived sleep measures, with comparison to a control population.

**Methods:**

A total of 241 dystonia cases were compared to 964 matched controls in analysis of self‐reported sleep symptoms and changes in sleep architecture using wrist‐worn triaxial accelerometers.

**Results:**

Dystonia participants had poorer self‐reported sleep patterns compared to controls. Accelerometery measurements demonstrated later sleep times, reduced time in bed, and shifts in circadian rhythm. No association was observed with pain, and only limited relationships with psychiatric symptoms.

**Discussion:**

This study demonstrates the utility of accelerometers in longer term evaluation of sleep in dystonia, for measurement of disturbance and response to treatment. Compared to controls, altered sleep and circadian rhythm were more common in dystonia patients which may contribute to the clinical phenotype.

## Graphical abstract

Accelerometer‐derived sleep measures in idiopathic dystonia: A UK Biobank cohort study. Grace A Bailey, Megan E Wadon, Sandra Komarzynski, Claare Matthews, Elin Haf Davies, Kathryn J Peall.* No previous studies have examined sleep in an idiopathic dystonia cohort using objectives measures derived from wrist worn accelerometers. We made us of the large UK Biobank dataset to undertake this work at scale. Data from 241 dystonia aand 964 matched controls were compared, identifying later sleep times, reduced time in bed and shifts in circadian rhythms in those diagnosed with dystonia compared to controls, with these differences independent of reported pain.

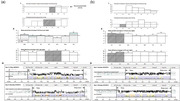

## 1 INTRODUCTION

Dystonia is a hyperkinetic movement disorder characterized by involuntary muscle contractions causing twisting, repetitive movements, and abnormal postures. Multiple non‐motor symptoms have been reported in association with dystonia, with sleep disturbance described in 40%–70% of idiopathic dystonia cohorts (Bailey et al., 2021; Hertenstein et al., 2016). However, to date, few studies have sought to examine sleep architecture through the use of objective measures such as laboratory‐based polysomnography (PSG). Those that have have demonstrated changes in participants with cranio‐cervical dystonia. Changes reported include a decrease in sleep efficiency (SE), total sleep time (TST) and percentage rapid eye movement (REM) sleep, and an increase in sleep latency (SL), wakefulness after sleep onset and REM SL, compared to controls (Antelmi et al., 2017; Lobbezoo et al., 1996; Ray et al., 2021; Samushiya et al., 2020; Sforza et al., 1991; Silvestri et al., 1990). Sleep fragmentation and increased REM SL have also been observed in *GCH1* mutation‐positive dopa‐responsive dystonia (Brüggemann et al., 2014), as well as higher numbers of sleep spindles observed in primary and secondary forms of dystonia (Fish et al., 1990).

Multiple factors influence sleep structure and quality, including pain and psychiatric symptoms. Both these traits that have been reported at higher than population levels in dystonia cohorts. However, the direction of the causal relationship in the interaction between these symptoms remains uncertain (Avanzino et al., 2010; Da Silva‐Júnior et al., 2021; Paus et al., 2011; Smit et al., 2017; Vu et al., 2021). Changes to sleep architecture, particularly REM sleep, are considered a distinctive neurological marker of depression, suggesting a complex bidirectional relationship between sleep and mental health disorders (Fang et al., 2019). Pain is also widely associated with impaired sleep quality and has been linked to sleep disturbances such as insomnia and reduced sleep duration. Excess pain is well recognized in dystonia and reported in up to 90% of those with cervical dystonia (Vu et al., 2021). It also appears to be a major contributor to impaired sleep quality amongst dystonia cohorts, with evidence of sleep impairment and pain severity being linked (Paus et al., 2011). There are no studies which have specifically investigated the relationship between self‐reports of pain and psychiatric symptoms, and actigraphy‐derived sleep variables in dystonia. Furthering our understandings of these relationships using a minimally intrusive monitoring system has the potential to impact the management of non‐motor symptoms in a primary care setting, particularly given the detrimental impact on an individual's quality of life.

Although laboratory‐based PSG remains the gold‐standard objective measure of sleep, accelerometer devices validated to detect sleep/wake patterns are increasingly being used to assess sleep parameters in the home environment and for extended periods of time (De Almeida et al., 2019; Gorbunov et al., 2020; Maglione et al., 2013; Maskevich et al., 2017). The UK Biobank (UKBB) is a large population‐based study integrating detailed clinical data, including raw accelerometer data, self‐reported symptom questionnaires, including pain and psychiatric symptoms, primary care records, and hospital admissions (Doherty et al., 2017). Here, we use UKBB actigraphy data to compare sleep disturbances and circadian rhythm across dystonia and matched controls and investigate correlations between actigraphy variables and self‐reported non‐motor symptoms, including pain and psychiatric symptoms. We hypothesize that sleep disturbances would be common in dystonia, relative to the comparison group, and that these relationships would be independent of confounding factors.

## 2 METHODS

### 2.1 Study cohort

Data was analyzed from the UKBB (MREC reference: 21/NW/0157) released to Cardiff University (project 13310), involving >500,000 adults (40–69 years) recruited throughout the UK (2006–2010). Individuals diagnosed with dystonia were identified using a previously validated algorithm (Bailey et al., 2021; Wadon et al., 2022), combining International Classification of Diseases version 10 (ICD‐10) codes and Read Code version 2 (Tables S1 and S2) (Bailey et al., 2021). The control cohorts were derived from participants matched for both age and gender on a 4:1 ratio (*n* = 964).

### 2.2 Non‐motor symptoms

Psychiatric, pain, and sleep disturbance symptoms were determined through a combination of diagnostic codes and symptom‐based questionnaires administered by the UKBB (Table S3). These codes were used due to their high completion rate within the dystonia cohort (>93%), and in keeping with previous studies (Carvalho‐e‐Silva et al., 2021; Fan et al., 2020; Kyle et al., 2017; Meng et al., 2020).

### 2.3 Accelerometer data

Physical activity was extracted from 100 Hz raw triaxial acceleration data obtained via Axivity AX3 wrist‐worn accelerometers in a total of 103,712 participants within the UKBB (2013–2015) (Doherty et al., 2017). Data was collected continuously over 7 days and processed and analyzed using R package GGIR (version 2.3‐0) (Migueles et al., 2019; Van Hees et al., 2018). Processing of data, including calibration, resampling and epoch generation has been described elsewhere (Doherty et al., 2017). Participants were excluded if they had fewer than 3 days recorded data (>16 h per day) or if they failed calibration (>0.01 g). Derived sleep features and physical activity (Table S4) measures were averaged across valid days.

### 2.4 Statistical analysis

Categorical data was compared between groups using Chi‐square test. Quantile plots were used to examine the normality of data, with parametric (Student's *t*‐test) or nonparametric (Mann–Whitney *U*) tests used for case–control comparison where appropriate, and Bonferroni correction applied for multiple comparisons. Associations among self‐reported sleep properties, physical activity, psychiatric diagnoses, pain symptoms, and accelerometery outcomes were assessed using linear regression analysis and adjusted for age at recruitment and sex.

## 3 RESULTS

The dystonia cohort (*n* = 1572) identified within the UKBB has been reported elsewhere (Wadon et al., 2022), of these, accelerometer data available for 241 (159 female, 82 male) unique individuals, and 15,768 age and sex‐matched controls (Table 1). Except for higher rates of daytime sleepiness in those with unspecified forms of dystonia (*p* = .007), no significant differences were observed across the self‐reported sleep symptoms. However, significantly poorer overall sleep patterns and lower sleep scores were seen in the overall cohort and those diagnosed with cervical dystonia (*p* < .001), compared to controls (Table 1).

**TABLE 1:** Demographics, self‐reported, and accelerometer‐derived sleep variables

		Dystonia	Blepharospasm	Cervical dystonia	Idiopathic familial dystonia	Idiopathic non‐familial dystonia	Idiopathic orofacial dystonia	Other dystonia	Dystonia, unspecified	Dystonic tremor	Control
Number		241	14	128	2	–	2	1	17	75	15,768
Age (mean, SD)		57.5 (7.9)	60 (8.18)	56.6 (7.6)	62 (0)	–	43 (1.4)	57	57.1 (9)	59 (8.1)	58.4 (7.8)
Age (range)		40–70	42–68	41–70	–	–	42–44	–	43–69	40–70	40–69
Gender	Male	82	4	47	0	–	0	0	4	27	4
	Female	159	10	81	2		2	1	13	48	13
		Dystonia	Blepharospasm	Cervical dystonia	Idiopathic familial dystonia	Idiopathic non‐familial dystonia	Idiopathic orofacial dystonia	Other/unspecified dystonia	Tremor	Control	
Sleep symptoms											
Insomnia		179/241 (74.3) 0.4	8/14 (57.1) 0.086	95/128 (74.2) 0.53	–	–	–	10/17 (58.8) 0.08	63/75 (84) 0.13	12,071/15,763 (76.7)	
Chronotype		89/218 (40.8) 0.09	6/13 (46.2) 0.41	46/115 (40) 0.3	–	–	–	6/15 (40) 0.7	29/68 (42.6) 0.21	5029/14,238 (35.5)	
Sleep duration		79/240 (32.9) 0.11	3/14 (21.4) 0.57	46/128 (35.9) 0.054	–	–	–	5/16 (31.25) 0.79	25/75 (33.3) 0.33	4443/15,727 (28.3)	
Snoring		83/225 (36.9) 0.22	5/12 (41.7) 0.52	52/124 (41.9) 0.035	–	–	–	5/14 (35.7) 0.83	18/70 (25.7) 0.2	4857/14,725 (33)	
Daytime sleepiness		9/241 (3.7) 0.1	0/14 (0) 0.58	4/128 (3.1) 0.47	–	–	–	2/17 (11.8) 0.007	3/75 (4) 0.28	343/15,740 (2.2)	
Sleep pattern											
Healthy		79/203 (38.9) 0.48	6/11 (54.5) 0.38	42/111 (37.8) 0.45	–	–	–	5/13 (38.5) 0.83	4/63 (6.3) 0.43	5500/13,920 (41.4)	
Intermediate		109/203 (53.7) 0.62	4/11 (36.4) 0.2	59/111 (53.2) 0.63	–	–	–	8/13 (61.5) 0.66	36.63 (57.1) 0.78	7366/13,290 (55.4)	
Poor		15/203 (7.4) 0.0008	1/11 (9.1) 0.27	10/111 (9) 0.0006	–	–	–	0/13 (0) 0.51	23/63 (36.5) 0.16	424/13,290 (3.2)	
Sleep score		3 (2–4) 0.05	4 (2–4) 0.83	3 (2–4) 0.02	–	–	–	3 (2–4) 0.78	3 (3–4) 0.32	3 (3–4)	
Accelerometery		Dystonia	Controls	*p*‐Value							
*Sleep*		241	964								
Bedtime		23:55 (01:14)	23:36 (01:16)	**.001**							
Wake‐up time		07:11 (01:41)	07:06 (01:17)	.42							
Time in bed (h)		7.24 (1.37)	7.45 (1.4)	**.002**							
Sleep duration (h)		6.5 (1.43)	6.71 (1.36)	.007							
Sleep efficiency (%)		90.5 (5)	90.26 (5.26)	.56							
WASO		0.69 (0.37)	0.74 (0.43)	.07							
Number of nocturnal awakenings		12.8 (4.67)	13.29 (4.5)	.39							
Number of daytime naps		8.47 (6.67)	8.3 (6.17)	.39							
Duration of daytime naps		1.55 (1.35)	1.52 (1.34)	.29							
Duration of longest sleep bout		8 (1.4)	8 (1.4)	.81							
*Circadian rhythm*		241	964								
L5 time		04:51 (06:38)	00:25 (01:14)	**<2.2e − 16**							
L5 acceleration (mg)		3.57 (1.39)	3.74 (1.97)	.011							
M5 time		10:23 (02:30)	03:47 (05:53)	**<2.2e − 16**							
M5 acceleration (mg)		58.7 (22.1)	59.19 (24.31)	.22							
*Physical activity*		240	964								
Overall physical activity (mg)		27.5 (10.15)	28.14 (10.55)	.1299							
Daytime acceleration (mg)		37.95 (14.47)	38.59 (15.03)	<2.2e − 16							
Sleep time acceleration (mg)		3.98 (1.53)	3.97 (1.68)	.8177							
Inactive time (min)		629.6 (149)	633.81 (130.74)	.84							
Light time (min)		258.77 (72.08)	260.02 (75.82)	.4							
Moderate time (min)		94.56 (60.61)	95.68 (58.74)	.9							
Vigorous time (min)		2.13 (2.9)	2.31 (3.73)	.17							
Bouts of 30 min inactivity		322.33 (202.11)	333.89 (193.37)	.93							
Bouts of 1‐min MVPA		38.36 (36.74)	36.71 (37.81)	.74							
Least active 5 h (mg)		3.74 (1.68)	3.53 (176)	.23							
Most active 5 h (mg)		58.77 (21.9)	60.35 (24.35)	.02							

*Note*: Sleep symptom values given as *n* (%), *p*‐value. *p*‐Values are all versus controls. Bold *p*‐values represent significant values post Bonferroni correction for multiple comparisons.

Abbreviations: mg, milligravity; MVPA, moderate‐vigorous physical activity; SD, standard deviation; WASO, wakefulness after sleep onset.

**FIGURE 1:**
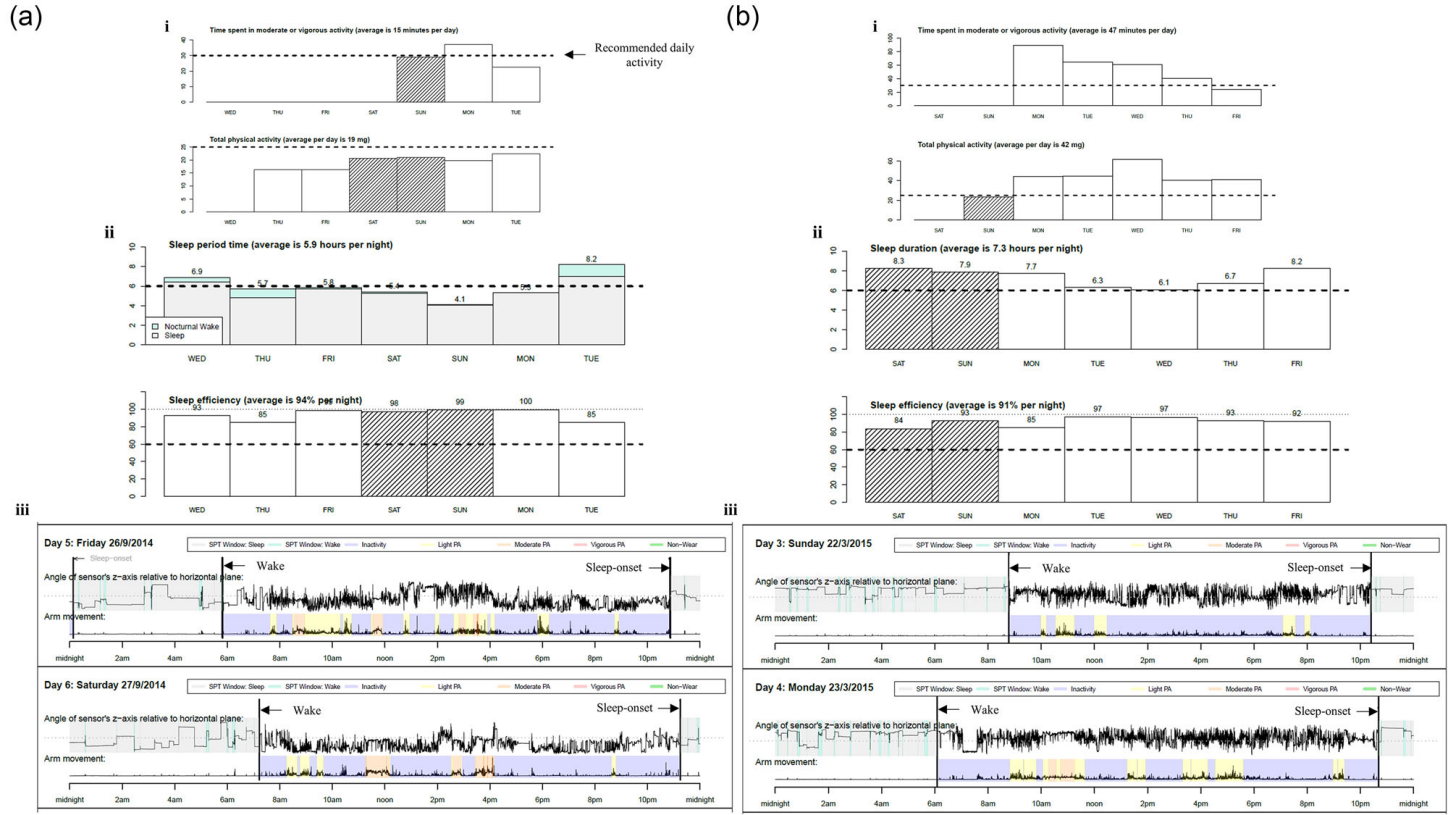
**Example of visual output reported by GGIR in the (A) dystonia and (B) control cohort. (i)** Bar plots with information on key physical activity (**ii)** bar plots with key/illustrative sleep variables (**iii)** visual summary of physical activity and sleep patterns. PA, physical activity; SPT, sleep period time.

Accelerometer activity was analyzed for the overall dystonia cohort owing to small cohort sizes within the individual subgroups (blepharospasm, *n* = 14; cervical dystonia, *n* = 128; familial, *n* = 2; orofacial, *n* = 2; other/unspecified, *n* = 18; tremor, *n* = 75; writer's cramp, *n* = 2) and controls. Individuals with dystonia were assigned to a single subtype of dystonia. Mann–Whitney *U* tests found that those with dystonia had a significantly later bedtime (*p* = .001) and spent less time in bed (TIB) (*p* = .002) compared to controls (Table 1). Compared to controls, individuals with dystonia had shifts and delays in circadian rhythm, least, and most active 5 h were later than controls (*p* < .001), although acceleration activity did not differ between groups. Those with dystonia were also less active during waking hours than controls, with reduced daytime acceleration (*p* < .001). Visual outputs reported by GGIR are shown in Figure 1.

Linear regression analysis found self‐reported sleep variables associated with their respective accelerometer‐derived sleep measures, including self‐reported chronotype and accelerometery‐derived sleep and wake time (*p* < .001 and *p* = .01), and self‐reported and accelerometery‐derived sleep duration (*p* < .001) (Table S5). Other associated variables included insomnia and sleep onset (*p* = .007), and nocturnal awakenings (*p* = .02) and number and duration of daytime naps (*p* < .001). Interactions between psychiatric diagnoses and the number and duration of daytime naps (*p* = .001 and *p* < .001, respectively) were also observed, alongside associations with accelerometer‐measured physical activity including daytime acceleration and earlier bedtimes (*p* = .001) and time spent being inactive and later bedtimes (*p* < .001). Daytime acceleration was associated with fewer and shorter daytime naps (*p* < .001), whereas durations of periods of inactivity were associated with reduced TIB (*p* < .001), TST (*p* < .001), and higher numbers of nocturnal awakenings (*p* < .001). There were no associations between self‐reported pain and accelerometer‐derived sleep variables.

## 4 DISCUSSION

This study represents the largest analysis of accelerometer‐derived sleep measures in dystonia to date. It also combines both the objective accelerometer measurements and subjective participant reported symptom measures to determine their reliability when determining disturbances to sleep in dystonia. Both measures demonstrated an excess of sleep disturbance in the dystonia cohort compared to controls, including higher rates of self‐reported poor sleep, and accelerometer determined reduced TIB and shifts in circadian rhythm. No association was observed between pain and sleep, and elevated psychiatric symptoms were only associated with a higher number of and longer daytime naps, suggesting that sleep disturbance is independent of these other non‐motor symptoms in dystonia. These findings, in contrast with previous literature, may in part be explained by the ‘healthy volunteer’ selection bias within the UKBB (Fry et al., 2017).

Several of the self‐reported sleep symptoms are consistent with previous studies, including poorer sleep patterns in the overall and cervical dystonia cohorts, whereas higher levels of daytime sleepiness were described by those with unspecified/other dystonia (Avanzino et al., 2010; Eichenseer et al., 2014; Han et al., 2020; Paus et al., 2011). In contrast, previous findings have shown high proportions of excessive daytime sleepiness and abnormal sleep duration amongst those with cranio‐cervical dystonia, potentially owing to use of standardized sleep questionnaires such as the Pittsburgh Sleep Quality Index and Epworth Sleepiness Scale (Avanzino et al., 2010; Paus et al., 2011). Further, the UKBB used only single questions for five aspects of sleep, potentially not allowing for the depth of symptom information needed to identify sleep disturbances.

Analysis of the accelerometer data found those with dystonia to have a significantly later bedtime and less time spent in bed compared to controls, in keeping with the findings from previous studies involving self‐reported sleep duration (Avanzino et al., 2010). A single PSG‐study to date has also reported increased SL in cervical dystonia, providing a potential explanation for the observed reduced sleep time (Antelmi et al., 2017).

Consistent with a single previously reported PSG‐based study, subjective self‐reported sleep variables were associated with their more objective, respective accelerometer measures, including sleep duration, chronotype, sleep onset, wake time, insomnia, and number of nocturnal awakenings (Antelmi et al., 2017). The domains in which less consistency was observed, such as self‐reported insomnia, SE, and TST, may in part be due to the long interval between baseline assessment and accelerometery data capture in some cases (average 5.9 years), as well as the well‐recognized differences that emerge between subjective and objective sleep measures (Jackowska et al., 2011; Van Den Berg et al., 2009).

In contrast to previous literature, we found no association between pain or psychiatric symptoms and accelerometer determined sleep disturbances. Several PSG and questionnaire‐based studies have shown that pain and mood disorders are strongly correlated with sleep impairment amongst dystonia cohorts (Eichenseer et al., 2014; Ray et al., 2021; Smit et al., 2017), although findings are inconsistent as to whether sleep‐related measures are independent of motor severity (Ray et al., 2021; Smit et al., 2017). It is possible that the small sample size (*n* = 13) amongst PSG‐based studies accounts for the association, whereas accelerometer data may provide a balance between accuracy and cohort size, and an area for future work.

In‐line with existing evidence, several associations between physical activity and accelerometer‐derived sleep measures were observed (Kredlow et al., 2015). Bouts of inactivity were significantly associated with later sleep time, reduced TIB, reduced TST and an increased number and duration of daytime naps. These findings suggest that physical activity can promote healthy sleep in those with dystonia, although we cannot rule out reverse causality.

Although this study benefits from the extensive longitudinal information collected at scale, several limitations also exist. First, the UKBB is a volunteer‐based sample and not representative of the general population (Fry et al., 2017), with participants potentially experiencing fewer symptoms that would interfere with participation. This is particularly relevant in the context of sleep disturbance as those who experience more severe dystonic symptoms may experience increased sleep impairments (Ray et al., 2021). Second, although the UKBB is a great resource for non‐motor symptom research, the reliability and validity of the measures remain largely unknown. For example, the validity of sleep behaviors in comparison with other established sleep questionnaires, such as the PSQI, has not been examined. Lastly, we were unable to confirm clinical diagnoses, and therefore, it remains possible that those without dystonia were included in the cohort. However, our cohort identification involved use of a previously published algorithm with 79% sensitivity in identifying those with dystonia (Bailey et al., 2021).

The present findings add to a growing body of evidence of sleep disturbances in dystonia, as well as demonstrating the capacity of accelerometery measurements in providing more detailed information about sleep patterns, beyond questionnaire‐based surveys. Our findings also suggest need for future investigation of physical activity and sleep in the context of dystonia, as well as the potential for the use of physical/exercise therapies as means for improved sleep quality in dystonia. Future work includes application of accelerometer‐based sleep measurements in larger dystonia cohorts, in conjunction with PSG‐based measurements, as well as longitudinal studies to examine whether sleep parameter variation is observed with varying motor symptom severity and therapeutic intervention.

## AUTHOR CONTRIBUTIONS

Conception; acquisition; execution; analysis and interpretation of the data; drafting and revising critically for important intellectual content: Grace A Bailey. Acquisition; execution; analysis and interpretation of the data; drafting and revising critically for important intellectual content: Megan E. Wadon. Acquisition; analysis and interpretation of the data; drafting and revising critically for important intellectual content: Sandra Komarzynski, Clare Matthews, Elin Haf Davies. Conception; acquisition; analysis and interpretation of the data; drafting and revising critically for important intellectual content: Kathryn J. Peall.

## CONFLICT OF INTEREST STATEMENT

The authors report no conflict of interests. SK and CM are employees at Aparito. EHD is employee at Aparito and holds shares.

## FUNDING INFORMATION

KESS2, European Social Fund and Cardiff University PhD Studentship in partnership with Aparito Limited; Jacques and Gloria Gossweiler Foundation; MRC Clinician‐Scientist Fellowship (MR/P008593/1)

### PEER REVIEW

The peer review history for this article is available at https://publons.com/publon/10.1002/brb3.2933.

## Supporting information

Table S1 ICD‐10 Codes and Read Codes used to identify dystonia patients.

Table S2 Exclusion codes (ICD‐10 and Read Codes) for UK Biobank participants.

Table S3 UK Biobank questions related to non‐motor symptoms and responses.

Table S4 Accelerometer‐derived sleep features and physical activity and their definitions.

Table S5 Associations between self‐reported sleep, pain/psychiatric symptoms and physical activity, and accelerometer‐derived sleep variables in the dystonia cohort.

## Data Availability

Anonymized data not published within this article will be made available by request by application to the UK Biobank (https://www.ukbiobank.ac.uk/enable‐your‐research/apply‐for‐access).
