# Supplementary material for: Accelerometer‐derived sleep measures in idiopathic dystonia: A UK Biobank cohort study
Source: Brain Behav. 2023 Aug 7;13(9):e2933. doi: 10.1002/brb3.2933 (PMC10498055; doi:10.1002/brb3.2933)
Supplement: Supplementary file 3 — Table S3 UK Biobank questions related to non‐motor symptoms and responses. [file BRB3-13-e2933-s005.docx]

**Supplementary Table 3.** UK Biobank questions related to non-motor symptoms and responses

| **Symptom** | **Description** | **Data Field** | **Question** | **Response** | **Risk** |
| --- | --- | --- | --- | --- | --- |
| Psychiatric | Mood swings | 1920 | Does your mood often go up and down? | Yes/No/Do not know/Prefer not to answer |  |
|  | Miserableness | 1930 | Do you ever feel ‘just miserable’ for no reason? | Yes/No/Do not know/Prefer not to answer |  |
|  | Irritability | 1940 | Are you an irritable person? | Yes/No/Do not know/Prefer not to answer |  |
|  | Fed-up feelings | 1960 | Do you often feel ‘fed-up’? | Yes/No/Do not know/Prefer not to answer |  |
|  | Nervous feelings | 1970 | Would you call yourself a nervous person? | Yes/No/Do not know/Prefer not to answer |  |
|  | Worrier/anxious feelings | 1980 | Are you a worrier? | Yes/No/Do not know/Prefer not to answer |  |
|  | Tense/’highly strung’ | 1990 | Would you call yourself tense or ‘highly strung’? | Yes/No/Do not know/Prefer not to answer |  |
|  | Worry too long after embarrassment | 2000 | Do you worry too long after an embarrassing experience? | Yes/No/Do not know/Prefer not to answer |  |
|  | Suffers from ‘nerves’ | 2010 | Do you suffer from ‘nerves’? | Yes/No/Do not know/Prefer not to answer |  |
|  | Loneliness/isolation | 2020 | Do you often feel lonely? | Yes/No/Do not know/Prefer not to answer |  |
|  | Guilty feelings | 2030 | Are you often troubled by feelings of guilt? | Yes/No/Do not know/Prefer not to answer |  |
|  | Seen doctor (GP) for nerves, anxiety, tension or depression | 2090 | Have you ever seen a general practitioner (GP) for nerves, anxiety, tension or depression? | Yes/No/Do not know/Prefer not to answer |  |
|  | Seen a psychiatrist for nerves, anxiety, tension or depression | 2100 | Have you ever seen a psychiatrist for nerves, anxiety, tension or depression | Yes/No/Do not know/Prefer not to answer |  |
| Pain | General pain for > three months | 2956 | Have you had pains all over the body for more than 3 months? | Yes/No/Do not know/Prefer not to answer |  |
|  | Pain type(s) experienced in last month | 6159 | Are you troubled by pain or discomfort, either all the time or on and off, that has been present for more than 3 months? | Headache  Facial pain  Neck or shoulder pain  Back pain  Stomach or abdominal pain  Hip pain  Knee pain  Pain all over the body  None of the above |  |
| Sleep | Sleep duration | 1160 | About how many hours sleep do you get in every 24 hours? (please include naps) | 1-23 hours, Do not know, Prefer not to answer | Low risk: 7-8 hours  High risk <7 hours or ≥9 hours |
|  | Morning/evening person (chronotype) | 1180 | Do you consider yourself to be? | Definitely a morning person, More a morning person than an evening person, More an evening person than a morning person, Definitely an evening person, Do not know, Prefer not to answer | Low risk: morning  High risk: evening |
|  | Insomnia/sleeplessness | 1200 | Do you have trouble falling asleep at night or do you wake up in the middle of the night? | Never/rarely, Sometimes, Usually, Prefer not to answer | Low risk: never/rarely  High risk: sometimes/usually |
|  | Snoring | 1210 | Does your partner or a close relative or friend complain about your snoring? | Yes, No, Do not know, Prefer not to answer | Low risk: no  High risk: yes |
|  | Daytime dozing/sleepiness (narcolepsy) | 1220 | How likely are you to doze off or fall asleep during the daytime when you don't mean to? (e.g. when working, reading or driving) | Never/rarely, Sometimes, Often, All of the time, Do not know, Prefer not to answer | Low risk: never/rarely or sometimes  High risk: often/all the time |
| Sleep pattern | Low risk summed, with higher scores indicating healthier sleeping patterns.  Healthy: ≥4  Intermediate: 2 or 3  Poor: ≤1 |  |  |  | Low risk = 1  High risk = 0 |
