# Supplementary material for: Accelerometer‐derived sleep measures in idiopathic dystonia: A UK Biobank cohort study
Source: Brain Behav. 2023 Aug 7;13(9):e2933. doi: 10.1002/brb3.2933 (PMC10498055; doi:10.1002/brb3.2933)
Supplement: Supplementary file 4 — Table S4 Accelerometer‐derived sleep features and physical activity and their definitions. [file BRB3-13-e2933-s003.docx]

**Supplementary Table 4.** Sleep features and physical activity and their definitions

| **Variable** | **Definition** |
| --- | --- |
| ***Sleep*** |  |
| Sleep onset | Median start time of sleep period, expressed as hours since the midnight of the previous night |
| Waking time | Median end time of sleep period, expressed as hours since the midnight of the previous night |
| Sleep duration | Total duration of sleep bouts during the primary sleep period |
| Time in bed | Difference between sleep onset and wake time |
| Sleep efficiency | Sleep duration/time in bed |
| Wake after sleep onset (WASO) | Duration of wake bouts during sleep period |
| Number of nocturnal awakenings | Number of wake bouts during sleep period |
| Number of naps | Number of sleep periods outside sleep period |
| Duration of longest sleep | Length of longest sleep bout during sleep period |
| ***Circadian rhythm*** |  |
| L5 time | Starting time of the least active five hours, expressed as timestamp in the day |
| L5 acceleration | Average acceleration over L5 |
| M5 time | Starting time of the most active five hours |
| M5 acceleration | Average acceleration over M5 |
| ***Physical activity*** |  |
| Overall physical activity | Average acceleration over 24-hour day in milligravity (mg) |
| Daytime physical activity | Average acceleration during the waking day in milligravity (mg) |
| Sleep time physical activity | Average acceleration during the sleep period in milligravity (mg) |
| Total inactive | Total minutes within waking hours spent inactive (<40mg) |
| Total light activity | Total minutes within waking hours spent in light activity (40-100mg) |
| Total moderate activity | Total minutes within waking hours spent in moderate activity (100-400mg) |
| Total vigorous activity | Total minutes within waking hours spent in vigorous activity (>400mg) |
| One-minute bouts of moderate-to-vigorous activity | Segments for which the acceleration within moderate-to-vigorous activity for one minute |
| 30-minute bouts of inactivity | Segments for which the acceleration is defined as inactive for 30-minutes |
| Least active five hours | Average acceleration during the five least active hours |
| Most active five hours | Average acceleration during the five most active hours |
