# Supplementary material for: Accelerometer‐derived sleep measures in idiopathic dystonia: A UK Biobank cohort study
Source: Brain Behav. 2023 Aug 7;13(9):e2933. doi: 10.1002/brb3.2933 (PMC10498055; doi:10.1002/brb3.2933)
Supplement: Supplementary file 5 — Table S5 Associations between self‐reported sleep, pain/psychiatric symptoms and physical activity, and accelerometer‐derived sleep variables in the dystonia cohort. [file BRB3-13-e2933-s001.docx]

**Supplementary Table 5.** Associations between accelerometer-derived sleep variables and self-reported sleep, pain/psychiatric symptoms and physical activity, in the dystonia cohort

| **Sleep variable** | **Sleep onset** | **Wake time** | **TIB** | **TST** | **SE** | **WASO** | **Number of nocturnal awakenings** | **Number of waking naps** | **Duration of daytime naps** | **Duration of longest sleep bout** |
| --- | --- | --- | --- | --- | --- | --- | --- | --- | --- | --- |
| **Sleep symptoms** |  |  |  |  |  |  |  |  |  |  |
| Sleep duration | -0.19  [-0.32, -0.06]  (0.00367 | **0.194**  **[0.07, 0.32]**  **(0.0029)** | **0.358**  **[0.24, 0.48]**  **(2.54e-08)** | **0.228**  **[0.18, 0.28]**  **(<2e-16)** | 0.046  [-0.08, 0.17]  (0.47) | 0.077  [-0.05, 0.2]  (0.23) | **0.296**  **[0.18, 0.42]**  **(2.06e-06)** | -0.11  [-0.23, 0.02]  (0.08) | **-0.096**  [-0.22, 0.03]  (0.1338) | **0.189**  **[0.06, 0.32]**  **(0.0039)** |
| Snoring | -0.02  [-0.15, 0.11]  (0.77) | 0.035  [-0.1, 0.17]  (0.60) | 0.049  [-0.08, 0.18]  (0.467) | 0.014  [-0.04, 0.07]  (0.61) | -0.063  [-0.19, 0.07]  (0.33) | 0.094  [-0.04, 0.22]  (0.15) | 0.008  [-0.12, 0.14]  (0.903) | -0.029  [-0.15, 0.10]  (0.65) | -0.023  [-0.15, 0.1]  (0.718) | 0.024  [-0.11, 0.16]  (0.722) |
| Daytime sleepiness | 0.015  [-0.11, 0.14]  (0.81) | 0.014  [-0.11, 0.14]  (0.83) | -0.005  [-0.13, 0.13]  (0.967) | -0.021  [-0.07, 0.03]  (0.406) | 0.032  [-0.09, 0.16]  (0.61) | -0.034  [-0.16, 0.09]  (0.59) | -0.058  [-0.18, 0.07]  (0.356) | 0.068  [-0.06, 0.19]  (0.28) | 0.114  [-0.01, 0.24]  (0.0693) | 0.116  [-0.01, 0.24]  (0.072) |
| Chronotype | **0.348**  **[0.22, 0.47]**  **(1.31e-07)** | **0.173**  **[0.04, 0.31]**  **(0.0105)** | **-0.186**  **[0.32, -0.05]**  **(0.0061)** | -0.067  [-0.12, -0.01]  (0.012) | 0.063  [-0.07, 0.19]  (0.406) | -0.099  [-0.23, 0.03]  (0.14) | -0.097  [-0.23, 0.03]  (0.141) | 0.114  [-0.02, 0.24]  (0.08) | 0.122  [-0.01, 0.25]  (0.0671) | -0.111  [-0.24, 0.02]  (0.103) |
| Insomnia | **0.173**  **[0.05, 0.3]**  **(0.00721)** | 0.08  [-0.05, 0.21]  (0.21) | -0.092  [-0.22, 0.04]  (0.156) | -0.069  [-0.12, -0.02]  (0.007) | -0.083  [-0.21, 0.04]  (0.192) | 0.046  [-0.08, 0.17]  (0.47) | **-0.141**  **[-0.26, -0.02]**  **(0.0242)** | **0.229**  **[0.11, 0.35]**  **(0.000108)** | **0.235**  **[0.11, 0.36]**  **(0.00016)** | 0.083  [-0.04, 0.21]  (0.2) |
| **Pain/psychiatric diagnoses** |  |  |  |  |  |  |  |  |  |  |
| Pain | -0.036  [-0.17, 0.09]  (0.58) | -0.027  [-0.16, 0.10]  (0.68) | 0.008  [-0.12, 0.14]  (0.9) | 0.007  [-0.12, 0.14]  (0.92) | -0.02  [-0.15, 0.11]  (0.76) | 0.004  [-0.12, 0.13]  (0.96) | 0.021  [-0.11, 0.15]  (0.75) | 0.075  [-0.05, 0.2]  (0.24) | 0.13  [0, 0.26]  (0.04) | 0.007  [-0.12, 0.14]  (0.92) |
| Psychiatric diagnosis | 0.12  [-0.01, 0.25]  (0.06) | -0.005  [-0.13, 0.12]  (0.94) | -0.121  [-0.25, 0.01]  (0.06) | -0.098  [-0.23, 0.03]  (0.14) | 0.037  [-0.09, 0.16]  (0.56) | -0.063  [-0.19, 0.06]  (0.32) | -0.113  [-0.24, 0.01]  (0.07) | **0.201**  **[0.08, 0.32]**  **(0.00131)** | **0.22**  **[0.1, 0.34]**  **(0.000437)** | -0.098  [-0.23, 0.03]  (0.135) |
| Psychiatric symptom | 0.069  [-0.06, 0.2]  (0.29) | -0.02  [-0.15, 0.11]  (0.73) | -0.086  [-0.21, 0.04]  (0.185) | -0.104  [-0.23, 0.02]  (0.11) | -0.093  [-0.22, 0.03]  (0.14) | 0.063  [-0.06, 0.19]  (0.32) | -0.073  [-0.2, 0.05]  (0.243) | 0.113  [0.01, 0.24]  (0.071) | 0.061  [-0.06, 0.19]  (0.33) | -0.104  [-0.23, 0.02]  (0.11) |
| **Physical activity** |  |  |  |  |  |  |  |  |  |  |
| Overall physical activity (mg) | -0.083  [-0.24, 0.02]  (0.107) | -0.133  [-0.26, 0.00]  (0.00429) | -0.019  [-0.15, 0.11]  (0.78) | -0.019  [-0.15, 0.11]  (0.779) | 0.012  [-0.12, 0.14]  (0.852) | 0.001  [-0.13, 0.13]  (0.98) | -0.015  [-0.14, 0.11]  (0.818) | **-0.389**  **[-0.50, -0.27]**  **(2.22e-10)** | **-0.372**  **[-0.49, -0.25]**  **(1.96e-09)** | -0.083  [-0.21, 0.05]  (0.212) |
| Daytime acceleration (mg) | **-0.211**  **[-0.34, -0.08]**  **(0.00127)** | -0.036  [-0.17, 0.09]  (0.58) | 0.17  [0.04, 0.3]  (0.01) | 0.169  [0.04, 0.3]  (0.0108) | 0.075  [-0.05, 0.2]  (0.246) | -0.01  [-0.14, 0.12]  (0.876) | 0.089  [-0.04, 0.21]  (0.164) | **-0.452**  **[-0.56, -0.34]**  **(7.62e-14)** | **-0.452**  **[-0.57, -0.34]**  **(1.34e-13)** | 0.062  [0.1, 0.2]  (0.354) |
| Inactive time (minutes) | **0.394**  **[0.28, 0.51]**  **(3.43e-10)** | -0.129  [-0.26, 0.00]  (0.0474) | **-0.495**  **[-0.61, -0.38]**  **(<9.39e-16)** | **-0.459**  **[-0.57, -0.34]**  **(1.51e-13)** | -0.124  [-0.25, 0.00]  (0.051) | -0.070  [-0.20, 0.06]  (0.275) | **-0.246**  **[-0.37, -0.13]**  **(7.45e-05)** | **0.588**  **[0.49, 0.69]**  **(< 2e-16)** | **0.608**  **[0.51, 0.71]**  **(<2e-16)** | **-0.269**  **[-0.39, -0.14]**  **(3.07e-05)** |
| Light time (minutes) | 0.014  [-0.12, 0.14]  (0.838) | -0.116  [-0.17, -0.07]  (0.146) | -0.10  [-0.23, 0.03]  (0.132) | -0.092  [-0.22, 0.04]  (0.164) | -0.013  [-0.14, 0.11]  (0.837) | -0.015  [-0.14, 0.11]  (0.812) | -0.051  [-0.18, 0.07]  (0.42) | **-0.443**  **[-0.56, -0.33]**  **(2.16e-13)** | **-0.378**  **[-0.49, -0.26]**  **(1.06e-09)** | **-**0.133  [-0.26, 0.0]  (0.044) |
| Moderate time (minutes) | -0.132  [-0.26, 0.00]  (0.0467) | -0.111  [-0.24, 0.02]  **(**0.095) | 0.026  [-0.11, 0.16]  (0.697) | 0.027  [-0.11, 0.16]  (0.692) | 0.019  [-0.11, 0.15]  (0.768) | -0.004  [-0.13, 0.12]  (0.954) | -0.005  [-0.13, 0.12]  (0.933) | **-0.293**  **[-0.41 -0.17]**  **(3.22e-06)** | **-0.290**  **[-0.41, -0.17]**  **(5.03e-06)** | -0.031  [-0.16, 0.1]  (0.642) |
| Vigorous time (minutes) | -0.042  [-0.17, 0.09]  (0.523) | 0.014  [-0.12, 0.14]  (0.836) | 0.054  [-0.08, 0.19]  (0.416) | 0.083  [-0.05, 0.21]  (0.214) | 0.103  [-0.02, 0.23]  (0.112) | -0.095  [-0.22, 0.03]  (0.144) | 0.081  [-0.04, 0.21]  (0.207) | **-0.178**  **[-0.3, -0.05]**  **(0.00525)** | **-0.194**  **[-0.32, -0.07]**  **(0.00251)** | 0.03  [-0.01, 0.16]  (0.652) |
| Bouts of 30 minutes inactivity | **0.336**  **[0.21, 0.46]**  **(1.85e-07)** | -0.024  [-0.15, 0.11]  (0.72) | **-0.342**  **[-0.47, -0.22]**  **(1.21e-07)** | **-0.307**  **[-0.43, -0.18]**  **(2.5e-06)** | -0.051  [-0.18, 0.08]  (0.43) | -0.085  [-0.21, 0.04]  (0.19) | **-0.185**  **[-0.31, -0.06]**  **(0.0035)** | **0.684**  **[0.459, 0.78]**  **(< 2e-16)** | **0.69**  **[0.60, 0.78]**  **(< 2e-16)** | -0.12  [-0.25, 0.01]  (0.069) |

**Abbreviations:** SE: Sleep efficiency, TIB: Time in bed, TST: Total sleep time, WASO: Wakefulness after sleep onset

Linear regression effects sizes (standardised beta coefficient), [confidence intervals (95%)], (p-value) for association between each accelerometer derived sleep measure and symptomatic sleep. Bold p-values represent significant associations as post Bonferroni correction for multiple comparisons (<0.005). Adjusted for sex and age.
